# An Exotic Long-Term Pattern in Stock Price Dynamics

**DOI:** 10.1371/journal.pone.0051666

**Published:** 2012-12-17

**Authors:** Jianrong Wei, Jiping Huang

**Affiliations:** Department of Physics and State Key Laboratory of Surface Physics, Fudan University, Shanghai, China; Universidad Veracruzana, Mexico

## Abstract

**Background:**

To accurately predict the movement of stock prices is always of both academic importance and practical value. So far, a lot of research has been reported to help understand the behavior of stock prices. However, some of the existing theories tend to render us the belief that the time series of stock prices are unpredictable on a long-term timescale. The question arises whether the long-term predictability exists in stock price dynamics.

**Methodology/Principal Findings:**

In this work, we analyze the price reversals in the US stock market and the Chinese stock market on the basis of a renormalization method. The price reversals are divided into two types: retracements (the downward trends after upward trends) and rebounds (the upward trends after downward trends), of which the intensities are described by dimensionless quantities, 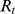 and 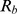, respectively. We reveal that for both mature and emerging markets, the distribution of either retracements 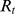 or rebounds 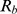 shows two characteristic values, 0.335 and 0.665, both of which are robust over the long term.

**Conclusions/Significance:**

The methodology presented here provides a way to quantify the stock price reversals. Our findings strongly support the existence of the long-term predictability in stock price dynamics, and may offer a hint on how to predict the long-term movement of stock prices.

## Introduction

Predicting the movement of stock prices has been regarded as one of the most challenging topics for modern scientists and economists. To understand the behavior of stock prices, a lot of properties of real stock markets have been reported and classified as so-called stylized facts [1], [2], such as volatility properties [3]–[5], correlation properties [5]–[9], scaling behavior [10], and fat tails in the probability distributions of log-returns [11], [12]. Also, numerous methods have been introduced to predict the price movement. Schoneburg (1990) analyzed the possibility of predicting prices of German stocks based on neural network approach [13]. Wang (2002) used fuzzy grey prediction system to predict the stock prices in Taiwan stock market [14]. Mittermayer (2004) showed that news textual analysis method can be used to forecast stock price trends [15]. Pai and Lin (2005) suggested a hybrid autoregressive integrated moving average model and support vector machines model in stock price forecasting [16]. It is worthy mentioned that, Kenett et al. (2012) recently reported that the average market correlation could be used as precursor to the changes in the stock market index [17].

On the other hand, there are also several existing theories which tend to render us the belief that the time series of stock prices are almost unpredictable on a long-term timescale. The efficient-market hypothesis developed by Fama (1970) believes that financial markets are informationally efficient. Even in its weak-form, the efficient-market hypothesis asserts that future prices cannot be predicted by analyzing prices from the past and thus no profitable information about future movement can be obtained in stock price series [18]–[20]. In the fields of physics and econophysics, the financial markets are sometimes considered as chaotic systems, in which a long-term prediction is impossible [21]–[24].

The question arises whether the long-term predictability exists in the stock price dynamics. In this work, we investigate the price reversals, the changes in the direction of price trends, in all 500 stocks of the S&P (Standard & Poor’s) 500 index. We divide the price reversals into two basis types: retracements and rebounds, which represent the downward trends after upward trends and the upward trends after downward trends, respectively. On the basis of a renormalization method, the intensities of retracements and rebounds are described by dimensionless quantities, 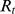 and 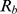, respectively. We reveal a bimodal distribution of both 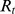 and 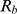, which indicates a long-term pattern in the stock price dynamics. In addition, we randomly reshuffle the price time series to test the robustness of the pattern. We also perform a parallel analysis in the Chinese stock market and obtain the similar pattern. This long-term pattern, which can be considered as one of the stylized facts in both mature and emerging markets, strongly supports the existence of the long-term predictability in the stock price dynamics, and may offer a hint on how to predict the long-term movement of stock prices.

## Methods

The direction of a price trend could be upward or downward. An upward trend means a series of increasing prices, while a downward trend means a series of decreasing prices. Naturally, the trend reversals, the changes in the direction of price trends, can be divided into two basis types: retracements (the downward trends after upward trends) and rebounds (the upward trends after downward trends). Here, both retracements and rebounds in discrete time series of stock prices are analyzed on the basis of a renormalization method.

**Figure 1:**
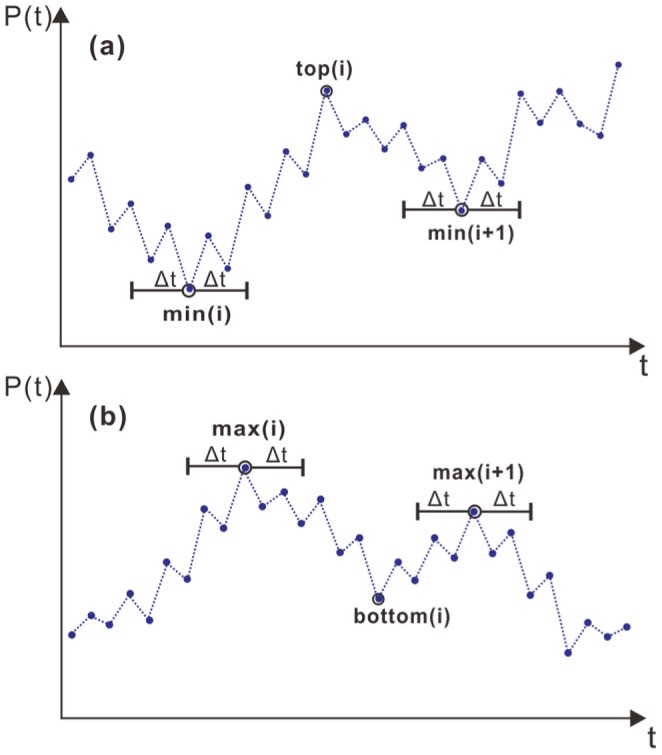
(Color online) Schematic graphs of (a) a retracement and (b) a rebound in a time series of stock prices. Here 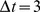 is taken as an example.

Denote the price at time *t* as 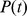, where 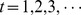. Each 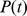 is defined to be a local minimum of 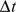 (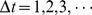) if there is no lower price in the time interval 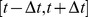 [25], [26]. In this way, all the local minima of a given 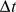 can be determined for the price time series [Fig. 1(a)]. These local minima are denoted by 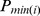, where 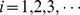. The highest price between two adjacent minima, 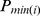 and 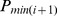, is represented by 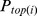. We introduce a renormalized retracement, 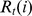, to describe the intensity of retracements as
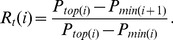
(1)


Here, to investigate the retracements of main trends, we focus on 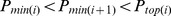, thus yielding 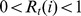. Clearly, a larger 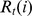 corresponds to a stronger retracement.

Analogously, to analyze the intensity of rebounds, each price 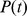 is defined to be a local maximum of 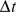 if there is no higher price in the time interval 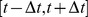 [25], [26]. We determine the local maxima of 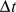 for the price time series [Fig. 1(b)], which are denoted by 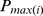. The lowest price between two adjacent maxima, 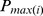 and 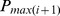, is denoted by 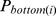. Then, the intensity of rebounds can also be described by a renormalized rebound, 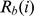, namely,
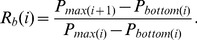
(2)Similarly, we focus on 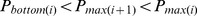, thus yielding 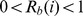. Here, a larger 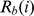 corresponds to a stronger rebound.

## Results

To investigate the stock prices in the US stock market, we analyze the time series of daily closing prices of all 500 stocks of the S&P 500 index. Totally, the time series comprise 2,768,341 closing prices which have been adjusted for dividends and splits. The oldest closing prices date back to January 2, 1962. The latest closing prices were recorded on April 24, 2012. It is noted that the lengths of price time series are different for different stocks in the analyzed database (see Table 1). The longest price series, IBM (International Business Machines Corporation), were recorded from January 2, 1962 to April 24, 2012, which contains roughly 12,000 prices. The shortest price series, NSM (Nationstar Mortgage), were recorded from March 8, 2012 to April 24, 2012, which contains only 33 prices.

**Table 1:** Samples of the analyzed database of the S&P 500 stocks.

Stock code	Corporation	Beginning date	End date	Number of days
IBM	IBM Corporation	January 2, 1962	April 24, 2012	12,666
YHOO	Yahoo! Inc.	Apirl 12, 1996	April 24, 2012	4,037
NDAQ	Nasdaq OMX Group Inc.	July 2, 2002	April 24, 2012	2,472
NSM	Nationstar Mortgage	March 8, 2012	April 24, 2012	33

**Figure 2:**
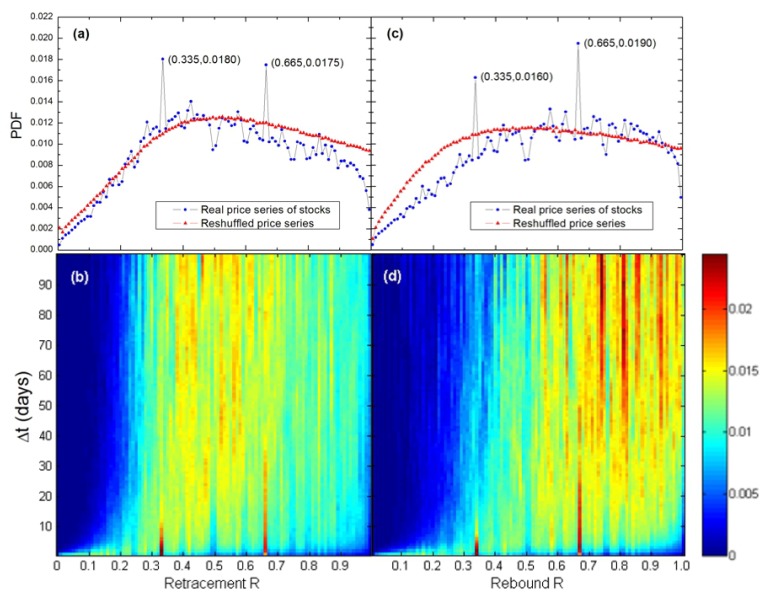
(Color online) PDF of (a,b) renormalized retracements and (c,d) renormalized rebounds of stock prices. (a,c) Overall PDF calculated over 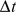 from 1 day to 100 days and over all trend reversals in the price series of all 500 stocks of the S&P 500 index. Each of them shows a bimodal distribution with two peaks located at two characteristic values of 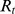 (or *R_b_*) = 0.335 and 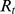 (or *R_b_*) = 0.665, which are symmetrical with respect to 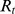 (or *R_b_*) = 0.500. Also shown are the results obtained from randomly reshuffled price series. (b,d) The colored PDF profiles. The color represents the probability density for each given 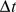. Ridges can be found at *R_t_* (or *R_b_*) = 0.335 and *R_t_* (or *R_b_*) = 0.665. The ridge of *R_t_* (or *R_b_*) = 0.335 stretches from 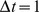 day to 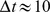 days. The ridge of *R_t_* (or *R_b_*) = 0.665 stretches from 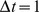 day to 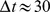 days.

In Fig. 2(a,c), the overall probability density functions (PDFs) of renormalized retracements and renormalized rebounds are calculated over 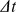 from 1 day to 100 days and over all trend reversals in the price series of all 500 stocks. Each of them shows a bimodal distribution with symmetrical peaks located at two characteristic values of 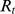 (or *R_b_*) = 0.335 and 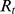 (or *R_b_*) = 0.665. These two peaks indicate that the probabilities of 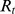 (or *R_b_*) = 0.335 or 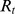 (or *R_b_*) = 0.665 are significantly higher than those of other values, suggesting a long-term pattern in the stock price reversals.

The colored PDF profiles are displayed in Fig. 2(b,d) where the color represents the probability density of retracements or rebounds for each given 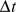. Two ridges located at *R_t_* (or *R_b_*) = 0.335 and *R_t_* (or *R_b_*) = 0.665 can be clearly found. Evidently, these two ridges mainly contribute to the two peaks in Fig. 2(a,c). As shown in Fig. 2(b,d), the ridges are sharper in the region of smaller 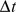, indicating this pattern in stock price dynamics attenuates as time span increases. This result is consistent with the common view that it is much harder to predict the stock prices on a longer time scale. It is noted that, in spite of their attenuation, the ridges can still be found on a rather long-term time scale. The ridge of 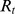 (or *R_b_*) = 0.335 stretches from 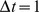 day to 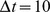 days. The ridge of 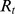 (or *R_b_*) = 0.665 stretches from 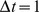 day to 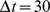 days.

To show the robustness of the pattern, we randomly reshuffle the price time series. Firstly, a log-return series is calculated from the original concrete time series of stock prices, 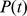, according to
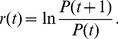
(3)Then we randomly reshuffle the log-return series. The reshuffled log-return series is denoted as 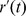. The new price time series is reproduced from the reshuffled log-return series according to
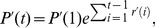
(4)with 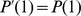. As shown in Fig. 2(a,c), the reshuffling yields the disappearance of the two peaks. Thus, the long-term pattern we have found is a consequence of price time series in the US stock market. It should be noted that, only one reshuffled PDF curve is shown in Fig. 2(a,c). The reason why we focus on one reshuffled PDF curve is two-folded. First, during the reshuffling process, all the price time series of 500 stocks of the S&P 500 index have been independently reshuffled, which means 500 independent reshuffles have been completed during one reshuffling process. Second, the overall PDF in Fig. 2(a,c) is calculated over 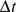 from 1 day to 100 days and over all trend reversals in the price series of all 500 stocks of the S&P 500 index, and thus the overall PDF itself is a kind of statistical average.

**Figure 3:**
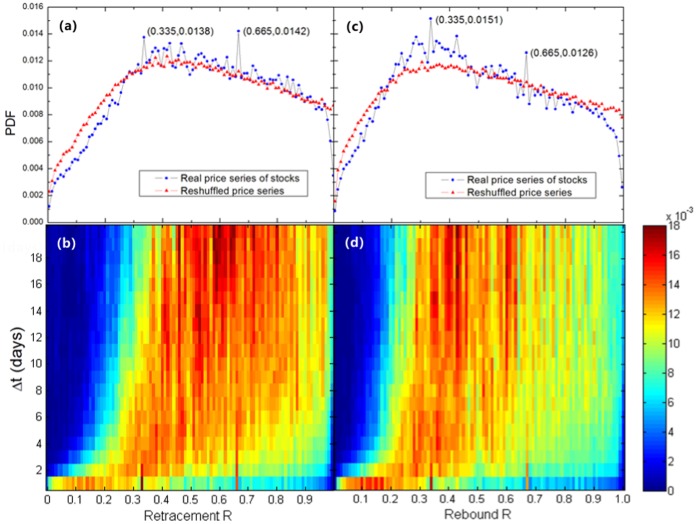
(Color online) The same as Fig.2, but for the Chinese stock market. (a,c) Overall PDF calculated over 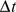 from 1 day to 20 days and over all trend reversals in the price series of all 300 stocks of the Shanghai Shenzhen CSI 300 index. Peaks can also be found at *R_t_* (or *R_b_*) = 0.335 and *R_t_* (or *R_b_*) = 0.665. (b,d) The colored PDF profiles. Ridges can also be found at *R_t_* (or *R_b_*) = 0.335 and *R_t_* (or *R_b_*) = 0.665.

We also perform a parallel analysis in the Chinese stock market, which is known as an emerging market. The database contains the daily closing prices of all 300 stocks of the Shanghai Shenzhen CSI (China Securities Index) 300 index from Jan 29, 1991 to May 7, 2012 (see Table 2). Similarly, Fig. 3(a,c) shows bimodal distributions of reversals in the Chinese stock market. Also, the peaks located at *R_t_* (or *R_b_*) = 0.335 and *R_t_* (or *R_b_*) = 0.665 can be found in the PDFs of renormalized retracements and renormalized rebounds. It should be noted that, because of the data limitation, a small range of 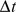 from 1 day to 20 days is taken during the analysis of the Chinese stock market. Consequently, the peaks located at *R_t_* (or *R_b_*) = 0.335 and *R_t_* (or *R_b_*) = 0.665 in Fig. 3(a,c) are not so sharp as those in Fig. 2(a,c). However, comparing with the PDF curves of reshuffled price series in Fig. 3(a,c), these two characteristic values can still be clearly obtained. Thus we suggest that the bimodal distribution of reversals is a universal pattern in both mature and emerging stock markets.

**Table 2:** Samples of the analyzed database of the Shanghai Shenzhen CSI 300 index stocks.

Stock code	Corporation	Beginning date	End date	Number of days
SZ000009	China Baoan	June 25, 1991	May 7, 2012	5,133
SH600031	SANY Heavy Industry	July 3, 2003	May 7, 2012	2,148
SH601857	PetroChina Company Limited	November 5, 2007	May 7, 2012	1,095
SH601288	Agricultural Bank Of China	July 15, 2010	May 7, 2012	437

## Discussion

This long-term pattern can be considered as one of the stylized facts in both mature and emerging stock markets. The reason for the emergence of the long-term pattern remains unknown. However, we suggest that this phenomenon should be due to the collective behavior of all traders in the stock markets. We also believe that agent-based modeling may have the merit to offer detailed insights into this exotic pattern [27]–[30]. This might serve as a future agenda of our research.

Furthermore, the result can be considered as a quantitative verifying of the empirical rules of so-called Fibonacci levels in stock markets [31], [32]. The technical traders, who use the Fibonacci levels in stock and future markets, believe that the intensity of a retracement (rebound) tend to be 0.382 or 0.618 times the intensity of the previous upward trend (downward trend). However, the existence of Fibonacci levels in real market has not been well verified. Here, we conclude that the specific levels do exist in the stock price reversals, although they are not exactly equal to the Fibonacci levels.

In summary, we have analyzed the reversals of daily stock prices in the US stock market and the Chinese stock market, and revealed that the distribution of either retracements or rebounds shows two symmetrical characteristic values, both of which are robust over long term. The peaks in the distributions of *R_t_* and *R_b_* indicate that the probabilities of *R_t_* (or *R_b_*) = 0.335 or *R_t_* (or *R_b_*) = 0.665 are significantly higher than those of other values, suggesting a long-term predictability in the stock price reversals. We also find that the ridges attenuate as time span increases, which is consistent with the common view that it is much harder to predict the stock prices on a longer time scale. Our findings suggest a long-term pattern, which strongly support the existence of the long-term predictability in the stock price dynamics and might offer a hint on the long-term prediction of stock prices. The methodology presented in this work also provides a way to quantify the price reversals in stock price dynamics.
